# Comment on the Article “A Lightweight and Low-Power UAV-Borne Ground Penetrating Radar Design for Landmine Detection”

**DOI:** 10.3390/s20103002

**Published:** 2020-05-25

**Authors:** Yuri Álvarez López, María García Fernández, Fernando Las-Heras Andrés

**Affiliations:** Área de Teoría de la Señal y Comunicaciones, Universidad de Oviedo, 33203 Gijón, Spain; garciafmaria@uniovi.es (M.G.F.); flasheras@uniovi.es (F.L.-H.A.)

**Keywords:** landmine detection, ground-penetrating radar, UAV, drone, synthetic aperture radar

## Abstract

This reply aims to correct some incomplete/incorrect information provided in the article “A Lightweight and Low-Power UAV-Borne Ground Penetrating Radar Design for Landmine Detection”, when the authors compare their results with some state-of-the-art contributions.

## 1. Background

The following Comment aims to correct some statements published in the article entitled “A lightweight and low-power UAV-borne ground-penetrating radar design for landmine detection” [1].

This article describes a simple, low-cost Stepped Frequency Continuous Wave (SFCW) radar whose size, weight, and power consumption make it suitable to be placed onboard an Unmanned Aerial Vehicle (UAV) for buried landmine detection.

The radar has been tested with ground tests (Section 5.1 of [1]), as shown in Figure 12 of [1], where a B-scan was conducted. Then, in Section 5.2, authors show measurement results with the radar onboard a UAV, comparing the performance with other airborne-based Ground Penetrating Radar (GPR) systems in Section 5.3.

This Comment is focused on the comparison presented in [1], where the authors compare the system in [1] and the one described in the publication [2]. The latter (reference No. 14 in [1]) is entitled “Synthetic Aperture Radar Imaging System for Landmine Detection Using a Ground Penetrating Radar on Board an Unmanned Aerial Vehicle”.

## 2. Corrections to the Comparison Published in the Article

The comparison between the systems described in [1] and [2] has some mistakes. The authors of this Comment, who have developed the system described in [2], would like to provide some comments and corrections, resulting in a fairer comparison.

First, all the results published in [2] are based on Synthetic Aperture Radar (SAR) processing. For example, Figure 12 of [2] compare airborne GPR images before and after SAR processing, yielding a better detection capability. The authors would like to remark that SAR processing is not applied in the UAV-based measurements shown in [1]. In fact, in [1], there is no information regarding their positioning systems and if they have the required positioning accuracy in performing SAR processing.

Second, Table 2 of [1] (entitled “Comparison of GPR system used for UAV purposes”) is not accurate when describing the features of the system shown in [2]. For example, in the last paragraph before Section 6 of [1], it is stated that:

“The radar module [29] of System 3 has lower power consumption, but as in the paper is stated an onboard computer (Raspberry Pi) is used to store acquired data.”

However, this is not correct, as the system described in [2] uses the Raspberry micro-computer for sending the measurements in real-time and also for controlling the UAV flight (i.e., an additional UAV flight controller is not used nor required). So, the statement of Table 2 of [1], “Power consumption of system 3: 2–3 W (only radar)”, is incorrect.

Third, concerning the results, the authors of [1] state that:

“System 3: on-ground tests have been performed in sandy soil, where a metallic disk was buried down to 15 cm. In-flight tests have included a sandbox, where a metallic disk was buried down to 12 cm. The sandbox was covered with a canvas.”

This statement is not correct. As it can be checked in [2], tests with buried plastic objects have been conducted. In particular, Figure 11 of [2] shows the 18 cm diameter plastic disk that was buried in sand, as well as the SAR images taken using the airborne GPR, where the plastic disk is clearly visible. As stated in [2]:

“SAR imaging also provides a substantial improvement over metal detector-based techniques [27], as non-metallic objects can be detected as well. To prove this feature, a plastic (foam) disk having the same radius [9 cm] as the metallic one, Fig. 11 (a), has been buried 10 cm deep. SAR image from coherent combination of the geo-referred GPR measurements collected during an overflight, considering ε_r_ = 1 for underground SAR imaging, are depicted in Fig. 11 (b). In this case, not only the air-sandbox interface and the plastic disk are imaged, but also the reflection created by the sandbox-ground interface is visible”.
